# High-Speed Incoming Infrared Target Detection by Fusion of Spatial and Temporal Detectors

**DOI:** 10.3390/s150407267

**Published:** 2015-03-25

**Authors:** Sungho Kim

**Affiliations:** Yeungnam University, 280 Daehak-Ro, Gyeongsan, Gyeongbuk 712-749, Korea; E-Mail: sunghokim@ynu.ac.kr; Tel.: +82-53-810-3530

**Keywords:** incoming target, target detection, stationary and moving, spatial and temporal, detector fusion, threat priority

## Abstract

This paper presents a method for detecting high-speed incoming targets by the fusion of spatial and temporal detectors to achieve a high detection rate for an active protection system (APS). The incoming targets have different image velocities according to the target-camera geometry. Therefore, single-target detector-based approaches, such as a 1D temporal filter, 2D spatial filter and 3D matched filter, cannot provide a high detection rate with moderate false alarms. The target speed variation was analyzed according to the incoming angle and target velocity. The speed of the distant target at the firing time is almost stationary and increases slowly. The speed varying targets are detected stably by fusing the spatial and temporal filters. The stationary target detector is activated by an almost zero temporal contrast filter (TCF) and identifies targets using a spatial filter called the modified mean subtraction filter (M-MSF). A small motion (sub-pixel velocity) target detector is activated by a small TCF value and finds targets using the same spatial filter. A large motion (pixel-velocity) target detector works when the TCF value is high. The final target detection is terminated by fusing the three detectors based on the threat priority. The experimental results of the various target sequences show that the proposed fusion-based target detector produces the highest detection rate with an acceptable false alarm rate.

## 1. Introduction

An active protection system (APS) is designed to protect tanks from a guided missile or rocket attack *via* a physical counterattack. High explosive antitank (HEAT) missiles should be detected and tracked for active protection using radar and infrared (IR) [1]. The first generation APS required detection algorithms to locate sub-sonic targets (under 340 m/s). Recently, the previous APS has moved to the next generation APS (NG-APS) to handle kinetic energy missiles, such as HEMi (over Mach 3–6) [2]. This is a challenging detection problem, because hyper-velocity missiles need to be detected at least 6 km form the target. Although radar and IR complement each other, this paper focuses on the IR sensor-based approach, because it can provide a high resolution angle of arrival (AOA) and detect high temperature targets.

**Figure 1:**
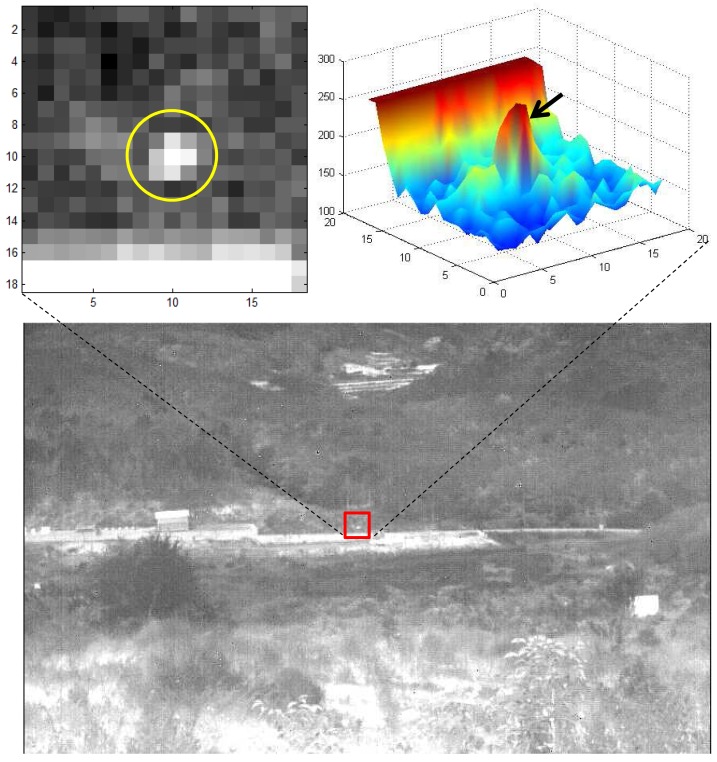
Infrared small target detection problem in a next generation active protection system (NG-APS).

In a real APS scenario, an incoming hyper-velocity target is shown as almost stationary in IR images at the firing stage and then moves slowly depending on the line of sight (LOS), as shown in Figure 1. In addition, small targets are located in the strong ground clutter. Therefore, it is a highly challenging problem to satisfy both the detection rate and false alarm rate simultaneously.

The previous small target detection method can be categorized into two approaches, spatial filter-based detection and temporal filter-based detection. Background subtraction can be a feasible approach if the target size is smaller than the background. The background image can be estimated from an input image using spatial filters, such as the least mean square (LMS) filter [3–5], mean filter [6], median filter [7] and morphological filter (top-hat) [8,9]. The LMS filter minimizes the difference between the input image and background image, which is estimated by the weighted average of the neighboring pixels. The mean filter can estimate the background by a Gaussian mean or simple moving average. The median filter is based on the order statistics. The median value can remove point-like targets effectively. The morphological opening filter can remove the specific shapes by erosion and dilation with a specific structural element. Mean filter-based target detection is computationally simple, but sensitive to thermal noise. Kim improved the mean subtraction filter by inserting a target enhancement and noise reduction filter, called modified MSF (M-MSF) [10]. Target detection using non-linear filters, such as the median or morphology filter, shows a low rate of false alarms around the edge, but the process is computationally complex. Combinational filters, such as max-mean or max-median, can preserve the edge information of background structures [11]. A data fitting approach that models the background as multi-dimensional parameters has also been reported [12]. The super-resolution method is useful in a background estimation, which enhances small target detection [13]. The filtering process of localized directional Laplacian-of-Gaussian (LoG) filtering and the minimum selection can then remove false detections around the background edges and maintain a small target detection capability [14].

Gregoris *et al.* introduced a target detection method based on the multiscale wavelet transforms for dim target detection [15]. Multiscale images can provide valuable structural information that can be used to distinguish the targets from clutter. Although it shows the feasibility of multiscale analysis for size varying target detection, it is computationally complex and cannot provide the precise size and location information. Wang *et al.* proposed an efficient method for the multiscale small target detection method by template matching [16]. They used a set of target templates to maximize the object-background ratio. A fast orthogonal search combined with a wavelet transform showed efficient small target detection performance [17]. Recently, Wang *et al.* proposed support vector machines in the wavelet domain and reported the feasibility of multiscale small target detection in low contrast backgrounds [18]. In addition, a multi-level filter-based small target detection method was implemented in real-time using DSP, FPGA and ASICtechnologies [19]. Size varying targets can be detected optimally via the scale-invariant approach using the signal-to-clutter ratio [20]. Qi *et al.* proposed Boolean map visual theory-based target detection by fusing the intensity and orientation information [21]. Although the method is effective in a cluttered environment, it incurs high computational cost and requires a huge memory allocation, because an input image is divided into many binary images in terms of the intensity and orientation channels.

Frequency domain approaches can be useful for removing low frequency clutter. The 3D-FFT spectrum-based approach shows a possible research direction in target detection [22]. The wavelet transform extracts the spatial frequency information in an image pyramid, which shows robustness in sea environments [23–25]. The low pass filter (LPF)-based approach can also be robust to sensor noise [26]. Recently, an adaptive high pass filter (HPF) was proposed to reduce clutter [27].

If a sensor platform is static, the target motion information is a useful detection cue. A well-known approach is the track-before-detect (TBD) method [28,29]. The concept is similar to that of the 3D matched filter. Dynamic programming (DP), which is a fast version of the traditional TBD method, achieves good performance in detecting dim targets [30,31]. The temporal profiles, including the mean and variance, at each pixel are effective in detecting moving targets in slow moving clouds [32–35]. Jung and Song improved the temporal variance filter (TVF) using the recursive temporal profile method [36]. Recently, the temporal contrast filter (TCF)-based method was developed to detect supersonic small infrared targets [37]. Accumulating the detection results of each frame makes it possible to detect moving targets [38]. The wide-to-exact search method was developed to enhance the speed of 3D matched filters [19]. Recently, an improved power law detector-based moving target detection method was presented; it was effective for image sequences that occur in heavy clutter [39]. Although the target is in motion, the previous frame is considered a background image. Therefore, a background estimation can be performed by a weighted autocorrelation matrix update using the recursive technique [40]. Static clutter can also be removed by the frame difference [41]. An advanced adaptive spatial-temporal filter derived from a multi-parametric approximation of clutter can achieve tremendous gain compared to that of the spatial filtering method [42]. Principal component analysis (PCA) for multi-frames can remove the temporal noise [43].

The above mentioned works showed their own pros and cons in the specific scenarios and environments. Despite this, no one has proposed a suitable small target detection method for an incoming target scenario in NG-APS. The key idea of this paper is to consider the incoming target behavior to maximize the detection rate and minimize the false alarms. The contributions of this paper can be summarized as follows. First, the motion of an incoming target is analyzed. Second, a multiple detector-based detection system is proposed to handle both stationary and moving targets. Third, a novel detector fusion method is proposed using the threat priority. Fourth, a computationally simple and effective method is proposed to cope with hyper-velocity targets.

This paper is organized as follows. Section 2 analyzes the target size and motion to find suitable detectors. Section 3 discusses the limitations of previous approaches. Section 4 introduces a new multiple detector fusion-based method. Section 5 explains various performance evaluations and results. The paper is concluded in Section 6.

## 2. Analysis of Incoming Targets in NG-APS

**Figure 2:**
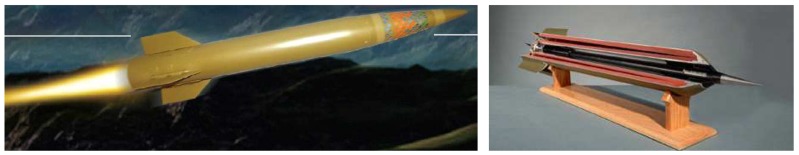
Examples of kinetic energy missiles.

A conventional APS aims to detect anti-tank missiles, such as RPG-7, Metis-M, Tow and Hellfire. Those missiles shows relatively slow speeds (around Mach 1). On the other hand, the recent trend is moving to a new anti-tank missile system, such as RPG-30 (the first fire is a decoy, and the second is the true target) or high kinetic energy missiles, like CKEMand HEMi, as shown in Figure 2. In particular, the kinetic energy missiles are more difficult to detect because of their hyper-speed (over Mach 6). The normal diameter is approximately 120 mm with a length of 1200 mm. The maximum missile range is around 5 km, and the missiles should be detected in at least 2 km to remove them.

**Figure 3:**
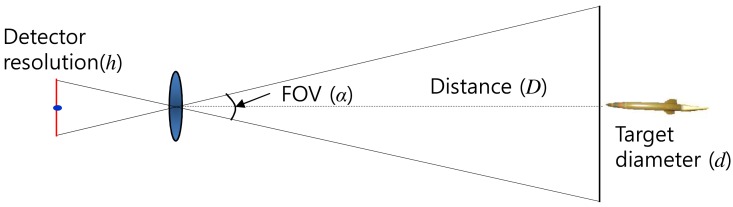
Target imaging geometry.

**Figure 4:**
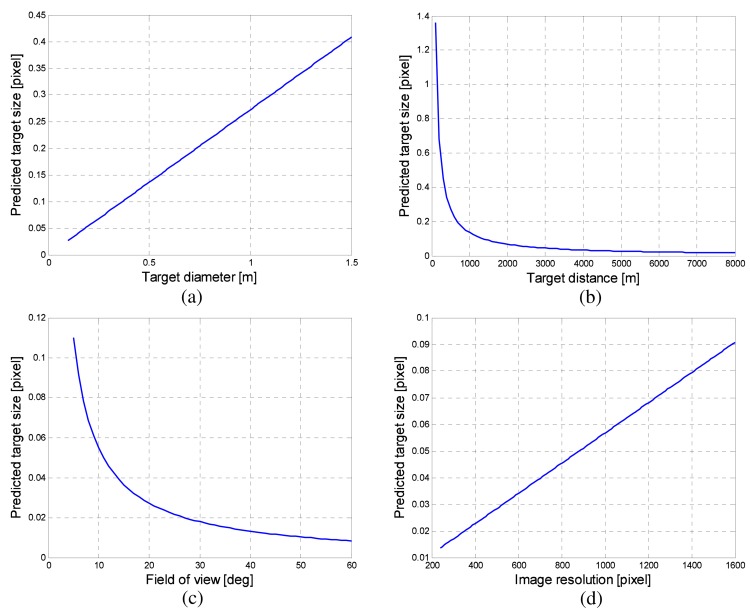
Analysis of an imaged target size depending on (**a**) target diameter; (**b**) target distance; (**c**) field of view and (**d**) image resolution.

The analysis of the imaged target size and motion behavior should be performed to predict the target size for optimal detector design. The imaged target size can be predicted using camera-target geometry, as shown in Figure 3. Let *h* denote the IR detector resolution, *α* denote the camera field of view (FOV) and *D* represent the target distance with the target diameter (*d*). The predicted target size (*x*) can be obtained using Equations (1) and (2). Figure 4 presents the analysis of the imaged target size depending on the target diameter (*d*), target distance (*D*), field of view (*α*) and image resolution (*h*). The default parameters are set as *h* = 480 pixels, *α* = 20°, *d* = 100 mm, and *D* = 5000 m. Each parameter was varied, while others have default values. The imaged target size is below one pixel considering the possible imaging scenarios. On the other hand, the thermal energy of a point target is dispersed (blurred) due to diffraction and aberration of the optical system [44]. Therefore, the actual target occupies several pixels, as shown in Figure 1.

(1) 2⋅D⋅tan(α/2):h=d:x

(2) $$x=\frac{h\cdot d}{2\cdot D\cdot \tan(\alpha /2)}$$

**Figure 5:**
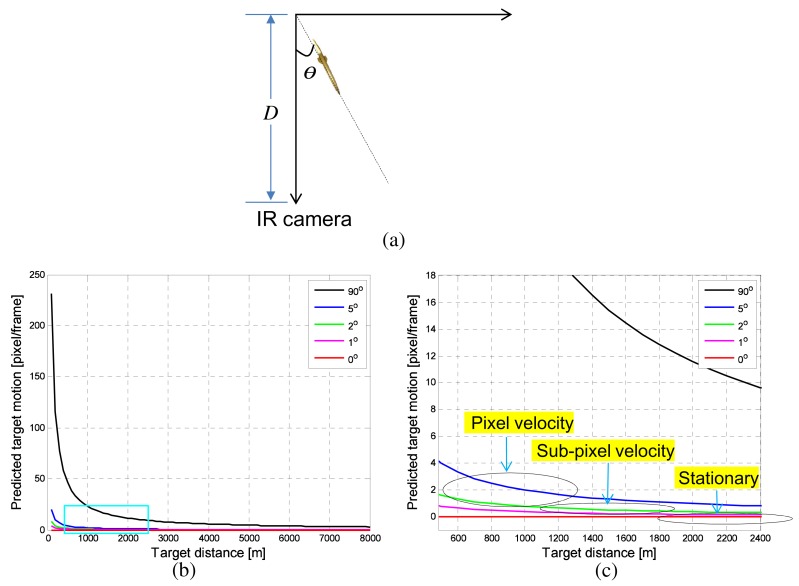
Analysis of imaged target motion depending on the incoming angle: (**a**) geometry of the incoming target (top-down view); (**b**) predicted target motion *vs.* the target distance (*D*); (**c**) enlarged graph.

The next analysis is target motion in an image according to the incoming angle (*θ*) and distance (*D*). If the geometry between an incoming target and IR camera is as shown in Figure 5a, the target motion (*L*) parallel to the image plane is defined as Equation (3) (m/frame). *S* denotes the target speed (ex. 340 × 6 (m/s)), and *f* represents the frames per second (ex. 120 Hz). The normal moving distance per frame is approximately 17 m/frame for a Mach 6 target. The target motion in an image with an incoming angle (*θ*) can be calculated using Equation (4). Figure 5b shows the predicted target motion per frame with respect to the target distance (*D*) and incoming angle (*θ*). *θ* = 0° represents the incoming motion of the line-of-sight (LOS) direction, and *θ* = 90° represents the passing-by target motion. Figure 5c shows an enlarged graph indicated by the rectangle region in Figure 5b. Note that the incoming target (0° < *θ* < 90°) shows different velocities, such as being stationary at a long distance, sub-pixel velocity at a mid-distance and pixel-velocity at a near-distance, depending on the incoming angle. The LOS target (*θ* = 0°) shows only stationary motion, and a passing-by target (*θ* = 90°) shows pixel-velocity motion. Other incoming angle targets show stationary, sub-pixel velocity and pixel-velocity.

(3) $$L=\frac{S}{f}\cdot \sin(\theta )$$

(4) $$l=\frac{h\cdot L}{2\cdot D\cdot \tan(\alpha /2)}$$

## 3. Problems of Previous Studies

### 3.1. 2D Spatial Filter: M-MSF

**Figure 6:**
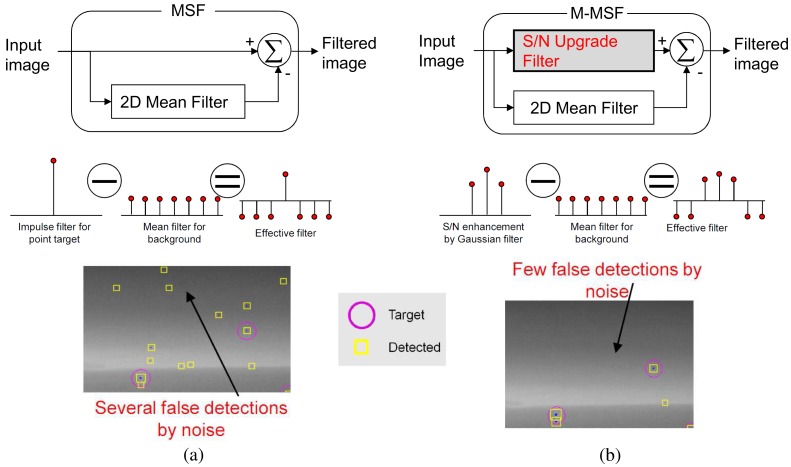
Basic concept of the modified mean subtraction filter (M-MSF): (**a**) previous MSF; (**b**) modified MSF by inserting an S/Nupgrade filter.

**Figure 7:**
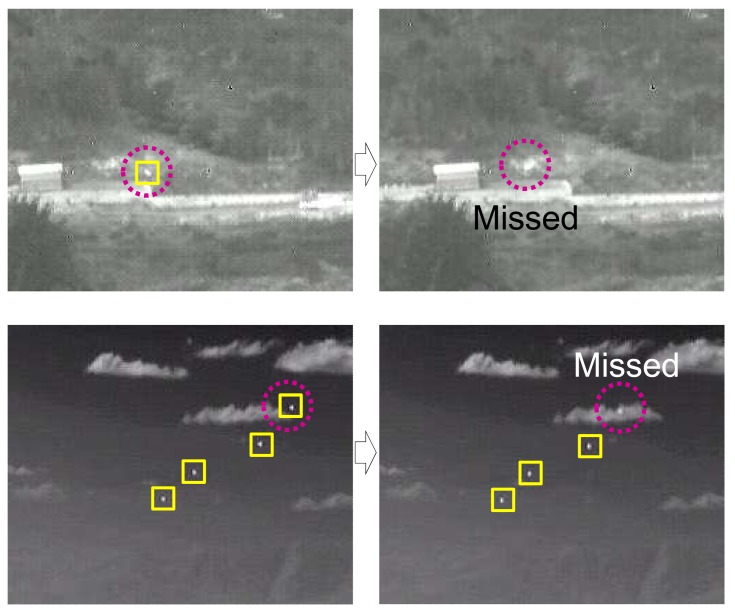
Limitation of the spatial filter (M-MSF)-based small target detection method.

The state-of-the-art spatial target detection methods are the scale-invariant (SI) method and Boolean map visual theory-based method (BMVT) [20,21]. SI shows the optimal detection in terms of the signal-to-clutter ratio for size varying targets. The method builds a 10–20 scale-space image and finds local extrema in space and scale. The BMVT constructs many Boolean maps for an intensity image and four orientation channels and weights each map based on statistics. The final targets are detected after fusing the intensity map and orientation map. Although these methods show the best performance in each specific area, they cannot be applicable to the proposed NG-APS, because of the heavy computational cost. The second best method is the modified mean subtraction filter (M-MSF) [10]. Figure 6 summarizes the basic concept of M-MSF by comparing with the previous MSF. The original MSF is a point target detector shown in Figure 6a, where an input image is subtracted from the background image. The MSF-based approach has been deployed in several countries because of its simplicity and the high detection capability of small targets [6,45]. The MSF, however, produces many false alarms around thermal noise or salt and pepper noise. The M-MSF modifies the original MSF by inserting an S/Nupgrade filter, as shown in Figure 6b. It is a 2D Gaussian filter with coefficient of [0.1 0.11 0.1; 0.11 0.16 0.11; 0.1 0.11 0.1], an approximated form of real infrared targets. The idea is similar to the matched filter theory to obtain the maximum signal-to-noise ratio. Therefore, the proposed M-MSF is conducted as follows. An input image (*I*(*x*, *y*)) is pre-filtered using the filter coefficients (*G*_3×3_(*x*, *y*)) to enhance the signal-to-clutter ratio (SCR), as shown in Equation (5) using the matched filter (MF). The SCR is defined as (max target signal − background intensity)/(standard deviation of the background). Simultaneously, the background image (*I_BG_*(*x*,*y*)) is estimated by a 7 × 7 moving average kernel (*MA*_7×7_(*x*, *y*)), as expressed in Equation (6). The pre-filtered image is subtracted by the background image, which produces an image (*I_M_*_−_*_MSF_*(*x*, *y*)), as shown in Figure 7.

(5) $$I_{MF}(x,y)=I(x,y)*G_{3\times 3}(x,y)$$

(6) $$I_{BG}(x,y)=I(x,y)*MA_{7\times 7}(x,y)$$

(7) $$I_{M-\text{MSF}}(x,y)=I_{MF}(x,y)-I_{BG}(x,y)$$

Although the M-MSF can provide a high detection rate with reduced false alarms, the spatial filter-based approach has a fundamental drawback when targets are located in a cluttered background, such as the ground or cloud, as shown in Figure 7. If targets are in ground clutter or cloud, the target shape is lost, which leads to a failure of the spatial target detection.

### 3.2. 1D Temporal Filter: TCF

**Figure 8:**
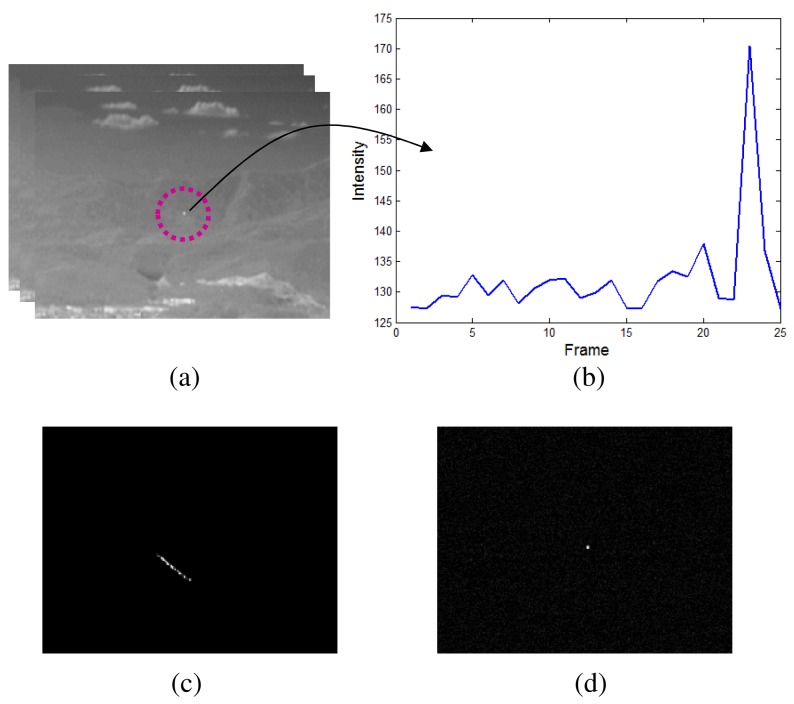
Basic concept of the temporal filter: (**a**) test sequence; (**b**) an example of temporal profile for a specific pixel; (**c**) temporal variance filter (TVF) result; and (**d**) temporal contrast filter (TCF) result.

The temporal profile-based moving target detection is effective in a cluttered environment. The well-known methods are the temporal variance filter (TVF) [46,47] and connecting line of the stagnation points (CLSP) [36,48]. If *I*(*i*, *j*, *k*) denotes the image intensity of pixel position (*i*, *j*) at the *k*-th image frame, the *TVF*(*i*, *j*, *k*) is defined by Equation (8) with a buffer size, *n* + 1. Because the TVF-based method detects the targets based on the stripe patterns, it shows high detection performance. On the other hand, it has limitations, such as the ambiguity of the target position and a sub-pixel velocity assumption, as shown in Figure 8c. The TCF can overcome the limitation by applying temporal contrast to the temporal profile, as shown in Figure 8b. The key idea of the TCF is the background signature estimation by the minimum filter to maximize the signal-to-noise ratio [37]. The *TCF*(*i*, *j*, *k*) is defined in Equation (9). The buffer size was assumed to be *n* + 1, and *n* frames were used to estimate the background intensity. As shown in Figure 8d, TCF can solve both the ambiguity of the target position and the target velocity.

(8) TVF(i,j,k)=var{I(i,j,k−n),I(i,j,k−n−1),⋯,I(i,j,k−1),I(i,j,k)}

(9) $$\text{TCF}(i,j,k)=I(i,j,k)-\underset{m=k-n,k-n-1,\cdots ,k-1,k-1}{\min}I(i,j,m)$$

**Figure 9:**
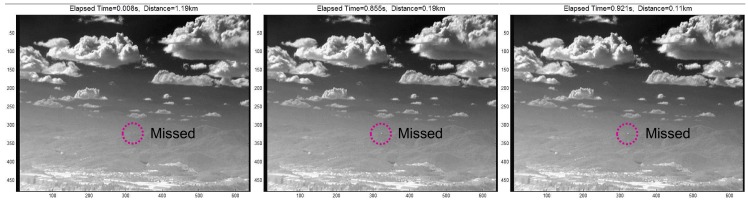
Limitation of the temporal filter (TCF)-based method small target detection method.

The TCF, however, cannot detect stationary targets, such as remote incoming targets or LOS incoming targets, as shown in Figure 9. The location and size of the incoming target has almost no change at both 1.19 km and 0.19 km. Therefore, a new target detection scheme is needed to compensate for the drawbacks of the spatial filter and temporal filter-based approaches.

## 4. Proposed Incoming Target Detection by Detector Fusion

As discussed in the previous section, M-MSF and TCF have pros and cons in terms of incoming target detection in a cluttered background. According to the motion analysis of incoming targets, the target speed in the image is almost stationary at the beginning. As time passes, the target shows a sub-pixel velocity and then a pixel-velocity. If the target comes directly (LOS), the speed in the image is almost zero. Therefore, neither method can work stably.

**Figure 10:**
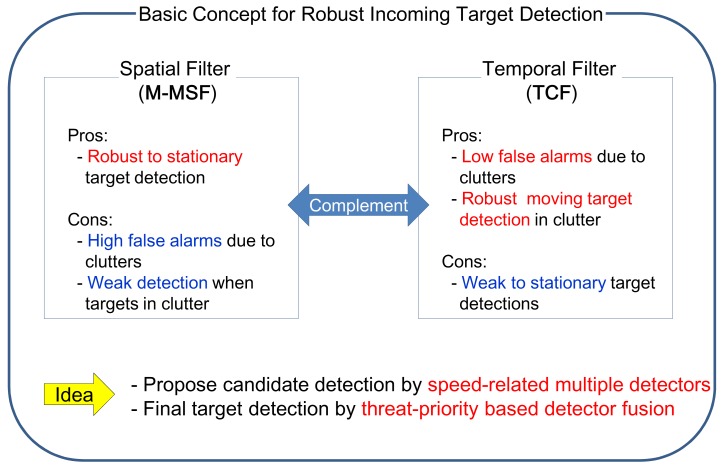
Basic idea for robust incoming target detection by the introduction of speed-related multiple target detectors and threat priority-based detector fusion.

The key idea stems from how to detect the incoming targets stably with a moderate false alarm rate. Figure 10 summarize the key idea. The spatial filter (M-MSF) and temporal filter (TCF) have their own advantages and disadvantages. Furthermore, the drawbacks of each method can be compensated using the other method. Therefore, the first idea is to find candidate targets using speed-related multiple detectors, and the second idea is to find the final targets using threat-priority-based detector fusion.

**Figure 11:**
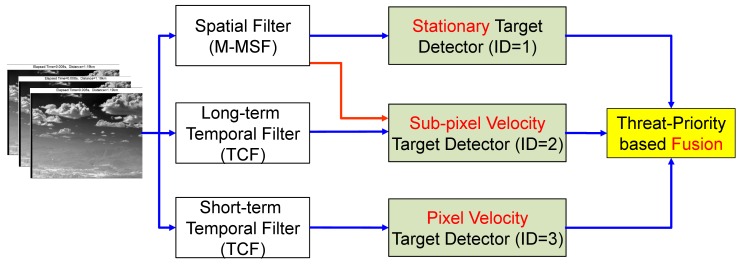
Proposed incoming target detection system. The system consists of basic filtering parts (M-MSF, TCF), speed-related detection parts and the detector fusion block.

Figure 11 represents the proposed incoming target system based on these concepts. At the top functional level, it consists of three parts: basic filters (M-MSF, TCF), speed-related detectors and detector fusion. Given an input sequence, an M-MSF and two TCFs with different buffer sizes (long-term and short-term) work in parallel. The stationary target detector produces almost no motion target using the M-MSF. The sub-pixel velocity target detector locates low speed targets using both long-term TCF and M-MSF. The pixel velocity target detector generates fast moving targets without spatial filter information. The candidate target information is merged in the threat-priority-based fusion.

### 4.1. Candidate Detection by Speed-Related Detectors

**Figure 12:**
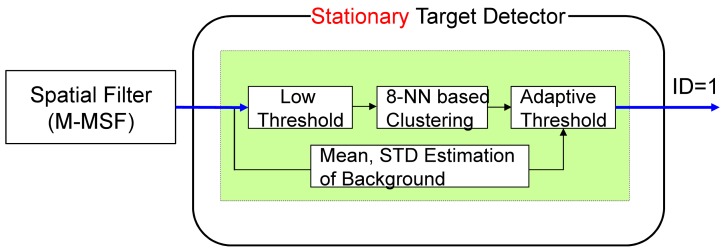
Stationary target candidate detector: given the M-MSF filtered image, spatial targets are detected using a hysteresis threshold constant false alarm (H-CFAR) detector.

**Figure 13:**
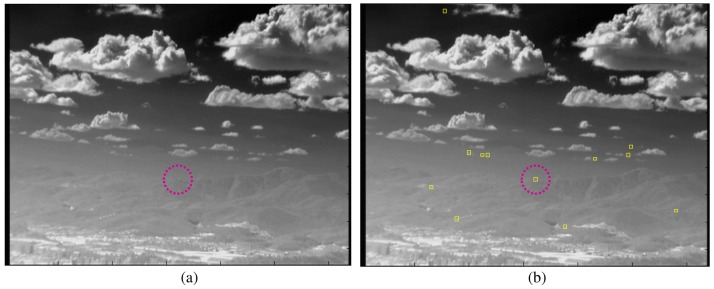
Example of stationary target candidate detector: (**a**) incoming target in line of sight (LOS); (**b**) stationary target detection results.

The stationary target detector consists of an M-MSF-based spatial target detector, as shown in Figure 12. The spatial targets are detected using a hysteresis threshold constant false alarm detector (H-CFAR) [49]. The pre-threshold (*Th_low_*) is selected to be as low as possible. The eight-nearest neighbor (8-NN)-based clustering method is used to group the detected pixels. The sizes of the possible targets can be estimated by 8-NN clustering. The probing region is divided into the target cell, guard cell and background cell. The target cell size is the same as the results of low threshold with clustering. The background cell size is determined to be three- to four-times the size of the target cell. The guard cell is just a blank region that is not used in both regions and is set as a two- or three-pixel gap. The second threshold (*Th_CFAR_*) in the CFAR can detect the final targets. *μ_BG_* and *σ_BG_* represent the average and standard deviation of the background region, respectively. *Th_CFAR_* controls the detection rate and false alarm rate. The output is target candidate locations with ID = 1. Figure 13 presents the results of stationary target candidate detection for an incoming target in LOS. In contrast to the TCF, the M-MSF-based stationary target detector produces the candidate regions successfully.

A probing region is a candidate target if:

(10) $$\text{SCR}(i,j)=\frac{\left|T_{\max}-\mu_{BG}\right|}{\sigma_{BG}}>Th_{\text{CFAR}}$$

**Figure 14:**
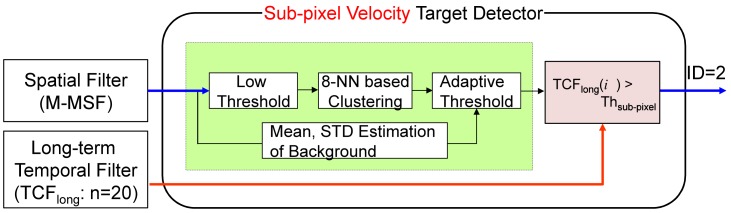
Sub-pixel velocity target candidate detector: given M-MSF filtered image, the spatial targets are detected using an H-CFAR detector. Slowly moving targets are detected using the long-term TCF information.

**Figure 15:**
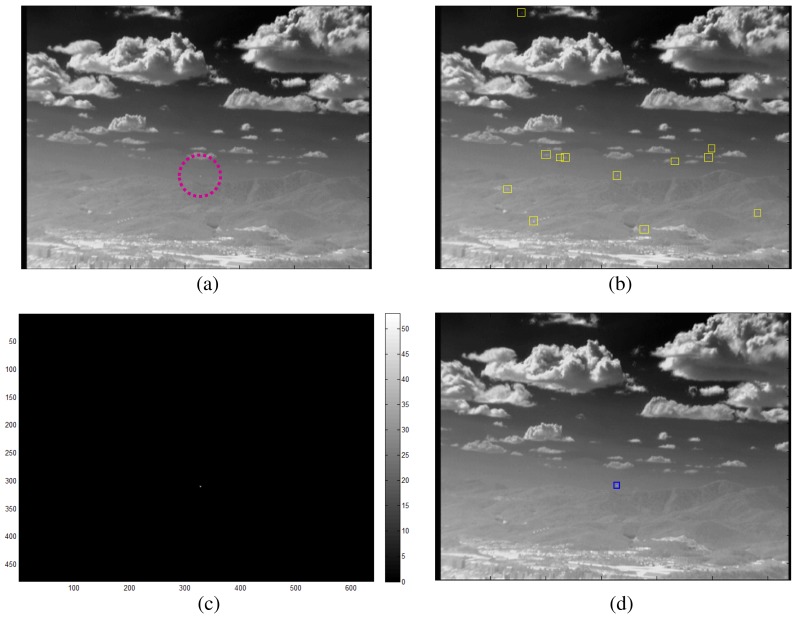
Example of a sub-pixel velocity target candidate detector: (**a**) incoming target (elevation angle: 2°, yaw angle: 1°); (**b**) M-MSF-based target candidate regions; (**c**) *TCF_long_* results; (**d**) results of the proposed sub-pixel velocity target detector.

The sub-pixel velocity (slow motion) target detector consists of an M-MSF-based spatial target detector and a long-term TCF-based speed checker, as shown in Figure 14. The long-term temporal filter (*TCF_long_*) is defined in Equation (9), where the buffer size (*n*) is approximately 20 frames to capture small target motion. The candidate target (position: (*i*, *j*)) detected by the spatial filter is confirmed if the *TCF_long_*(*i*, *j*) satisfies the following equation. Therefore, the second detector can generate slowly moving targets considering both motion and shape. The output is target candidate locations with ID = 2. Figure 15 shows the detection processing flow of the sub-pixel velocity target. The synthetic target is inserted to move slowly in the image (elevation angle: 2°, yaw angle: 1°), as shown in Figure 15a. Details for this are provided in the experimental section. The M-MSF-based spatial target detector can produce possible target regions (Figure 15b), and the *TCF_long_*-based temporal target detector can indicate the small motion pixels (Figure 15c). The spatial location and temporal motion information are combined and produce the final sub-pixel velocity target, as shown in (Figure 15d). Note that the false detections caused by the backgrounds (cloud, ground) can be removed effectively.

A probing region is the sub-pixel velocity target if:

(11) $${\text{TCF}}_{\text{long}}(i,j)>Th_{\text{sub}-\text{pixel}}$$

**Figure 16:**
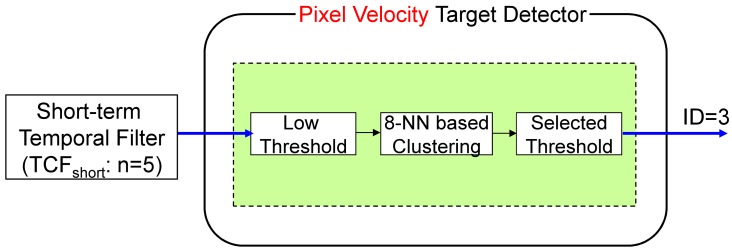
Pixel velocity target candidate detector: fast moving (pixel velocity) targets are detected using the short-term TCF and hysteresis thresholding.

**Figure 17:**
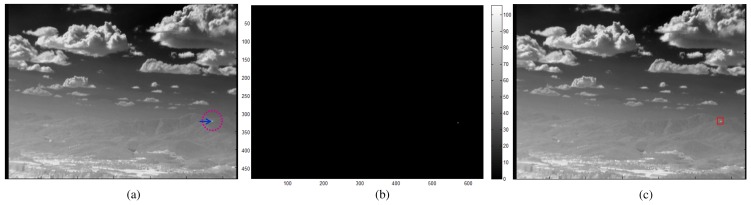
Example of the pixel velocity target candidate detector: (**a**) a passing-by target (elevation angle: 0°, yaw angle: 90°); (**b**) the processing results of *TCF_short_*; (**c**) the results of the proposed pixel velocity target detector.

The third detector is the pixel velocity detector, which can produces fast motion targets using the short-term temporal filter (*TCF_short_*) and hysteresis thresholding, as shown in Figure 16. The normal buffer size (*n*) is approximately five to capture fast moving targets. The hysteresis thresholding-based detection is quite simple, but can localize candidate targets. The output is the target candidate locations with ID = 3. Figure 17 shows the detection flow of the pixel velocity target detector. A sequence is generated by passing-by a target whose velocity is approximately eight pixels/frame (Figure 17a). A *TCF_short_* filter generates a pixel map of a fast moving target (Figure 17b). Final detection results ((Figure 17c)) are obtained by applying hysteresis thresholding to the fast motion map.

### 4.2. Final Detection by Detector Fusion

Recently, a psychologist reported synergistic interaction between temporal and spatial expectation in enhanced visual perception [50]. The information of temporal expectation can bias (modulate) the target perception. Similarly, the spatial or receptive field information can bias (modulate) the temporal expectation. In the proposed method, an M-MSF-based spatial detector and *TCF_short_*-based temporal detector work independently. The second detector, sub-pixel velocity target detector, utilizes both the spatial and temporal information.

According to the recent sensor and data fusion theory, data fusion is used to enhance target detection performance [51]. In terms of image processing, there can be pixel-level fusion, feature-level fusion and decision-level fusion. Pixel-level fusion is usually applied to different sensor images, such as infrared and visible or infrared and synthetic aperture radar (SAR) to enhance target pixels and reduce clutter pixels [52]. It is possible to encounter precise image registration problems with fusion data from different sensors. In feature-level fusion, features are extracted for each sensor and combined into a composite feature, representative of the target in the field. The SAR feature and infrared feature can be combined to enhance target detection or classification [53]. The features can be selected and combined to form a composite vector that can be trained and tested in classifiers, such as SVM and random forest. Decision-level fusion is associated with a sensor or channel. The results of the initial target detection by the individual sensors are input into a fusion algorithm.

Our system uses the framework of the decision-level fusion, because three independent channels can detect velocity-related targets, such as stationary, sub-pixel velocity and pixel velocity. In decision-level fusion, simple AND operation-based fusion can be a feasible solution. Tao *et al.* used the AND/OR rule to fuse a 2D texture image and 3D shape, which produced a reduced error rate [54]. Toing *et al.* found moving targets by applying the AND rule to fuse the temporal and spatial information [55]. Jung and Song used the same AND fusion scheme to fuse spatial and temporal binary information [36]. Fusing data collected from different sensors requires measurement accuracy (reliability or uncertainty), so that they can be fused in a weighted manner. The mathematical formulation can be Bayesian inference, fuzzy logic and belief theory (Dempster–Shafer model) [56]. The Bayesian inference (e.g., particle filtering) method computes the probability that an observation can be attributed to a given hypothesis, but lacks in its ability to handle sensor uncertainty [57]. Fuzzy logic methods use imprecise states and variables and can provide tools to deal with observations that are not easily separated into discrete segments [58]. The belief theory generalizes Bayesian theory to relax the restriction on mutually exclusive hypotheses, so that it is able to assign evidence to unions of hypotheses. Kumar *et al.* used the belief theory to fuse visible and infrared video for surveillance [56]. The precise belief modeling and sensor reliability assessment are the key part of the fusion algorithm.

**Figure 18:**
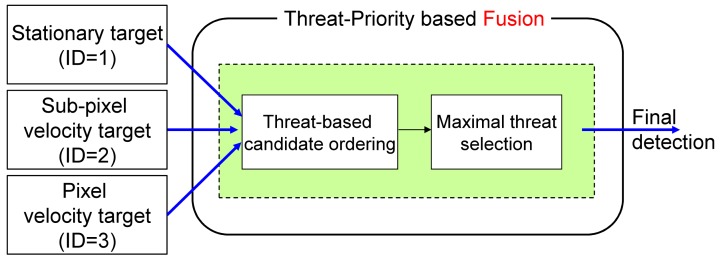
Threat-priority-based detector fusion using threat ordering and max selection.

In NG-APS, it is very important to achieve a high detection rate with moderate false alarms for successful protection. Each detector has its own pros and cons. The logical AND fusion can reduce false detections, but causes reduced target detection performance. On the contrary, the logical OR fusion can enhance the target detection rate with increased false detections. Note that both the AND and OR fusion schemes ignore target attribute information (velocity type) during fusion. In the proposed detector bank, each detector can find different kinematic targets, such as stationary, slow moving or fast moving motion. Therefore, weight-based decision fusion is not feasible in this incoming target scenario. If three detectors produce candidate targets, the fusion system merges the information in the decision level, as shown in Figure 18. In this paper, a new information fusion strategy, called max fusion, was proposed using threat-priority-based order statistics, as Equation (12). $\left[ID^{k},{\left(x,y\right)}^{k},V_{T}^{k}\right]$ represents the *k*-th target attributes, such as target type (*ID^k^*), position ((*x*,*y*)*^k^*) and threat level ( $V_{T}^{k}$). $D_{id=i}^{k}(x,y)$ denotes the threat of the *k*-th target detected at (*x*,*y*) in the *i*-th detector as Equation (13). The threat level of a candidate target can be defined depending on the scenarios. This paper uses a target velocity $\left(\upsilon_{i}^{k}(x,y)\right)$ in an image as a quantitative threat measure, because fast moving targets in an image are usually located near the camera sensor, as shown in the motion analysis (Figure 5).

(12) $$\left[ID^{k},{\left(x,y\right)}^{k},V_{T}^{k}]=\underset{id}{\max}\{D_{id=1}^{k}(x,y),D_{id=2}^{k}(x,y),D_{id=3}^{k}(x,y)\right\}$$

(13) $$D_{id=i}^{k}(x,y)=\upsilon_{i}^{k}(x,y)$$

**Figure 19:**
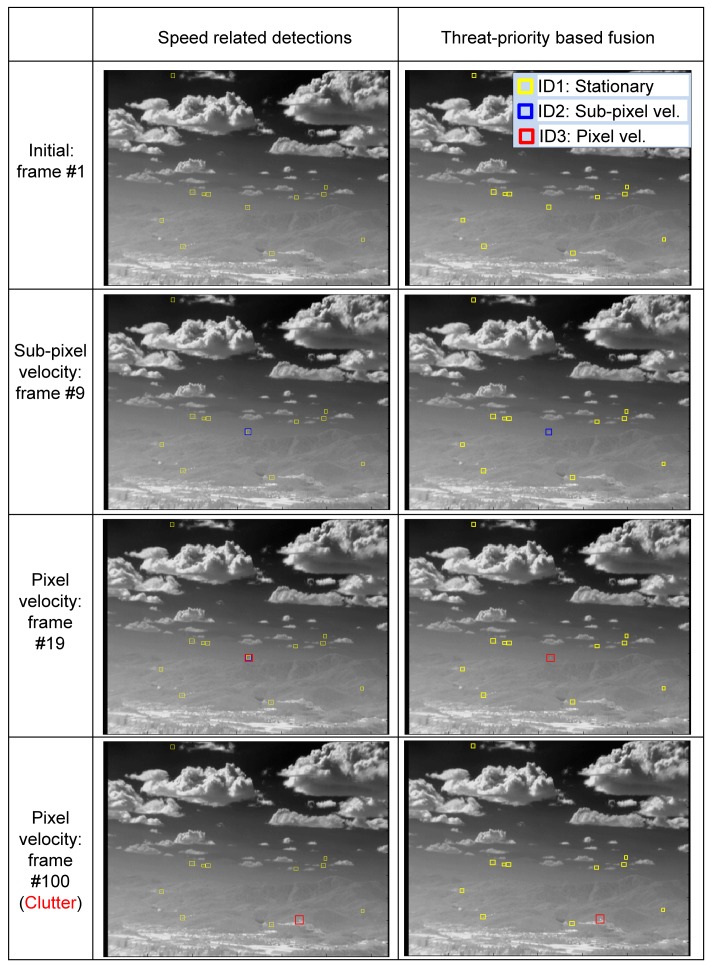
Examples of target detection using the proposed threat-priority-based detector fusion: (**Left**) before detector fusion; (**Right**) after detector fusion.

The proposed threat-priority-based fusion system can provide not only the target location, but also the threat level of the detected target. Figure 19 presents the effects of detector fusion for an incoming target. At the initial stage, only the spatial filter works (ID1: yellow square). After several frames, both the M-MSF (spatial filter) and *TCF_long_* (temporal filter) are activated. ID2 is selected according to the threat-priority. At the 19th frame, three detectors produce candidate targets, and the proposed fusion system selects ID3, which shows the highest threat. In the last row (100th frame), the target enters the strong ground clutter. Therefore, the stationary target and sub-pixel velocity detector do not work. The third pixel-velocity detector can produce a moving target that is selected in the fusion system.

## 5. Experimental Results

**Figure 20:**
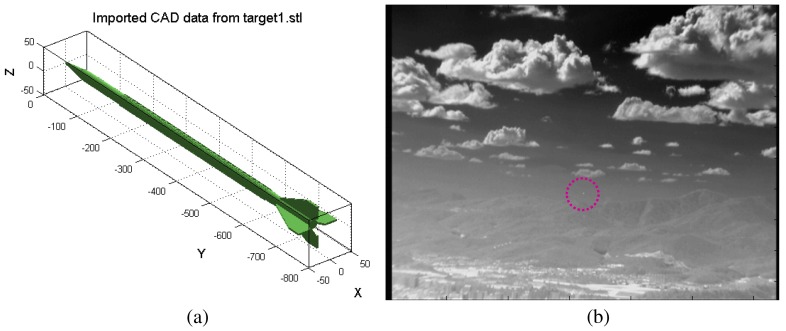
Test DB generation by inserting 3D CAD model into a real IR sequence: (**a**) 3D target CAD model; (**b**) synthesized result: target distance 1.2 km, target radiation: 50 W/sr.

A test database needs to be prepared to validate the proposed target detection method. The acquisition of an incoming target in the LOS direction is almost impossible. Therefore, two kinds of synthetic test sequence generation methods and real IR camera-based incoming target sequences were prepared. The first synthetic sequence generator is based on the synthesis of a 3D target model to a real IR image background using physics-based geometric and radiometric modeling [44]. The direction of the incoming target can be configurable using the elevation angle and yaw angle. The reference line is the LOS between the target and camera. Therefore, the elevation angle (0°) and yaw angle (0°) represent direct incoming in the LOS direction. Figure 20 shows the 3D target model and its synthesized result. The synthesized target is very small at a 1.2-km distance. The LOS incoming sequence (Set1-elevation: 0°, yaw: 0°), passing-by sequence (Set2-elevation: 0°, yaw: 90°) and a general incoming sequence (Set3-elevation: 2°, yaw: 1°) with the target speed of Mach 3 were generated.

**Figure 21:**
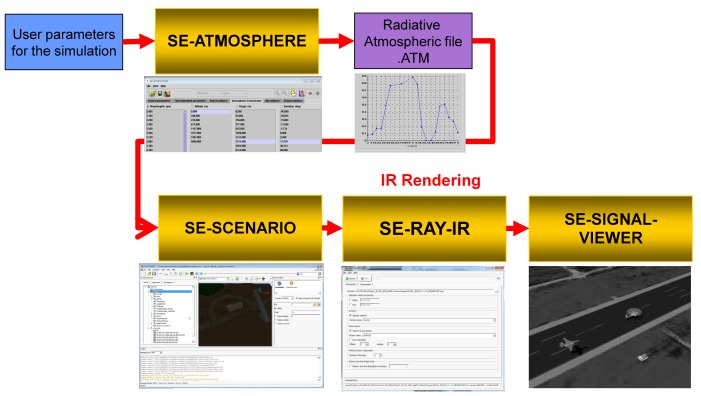
The flow of synthetic IR image generation using OKTAL-SE.

**Figure 22:**
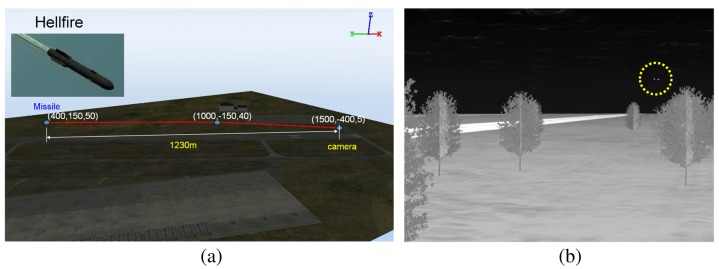
OKTAL-SE-based incoming target generation: (**a**) geometric scenario of incoming target using the Hellfire missile; (**b**) mid-wave IR image generation.

For the second synthetic sequence generator, commercial software, called OKTAL-SE, was used [59]. OKTAL-SE is the only simulator that can synthesize both passive (IR) and active (synthetic aperture radar) data. Figure 21 summarizes the overall flow of IR synthesis. Given simulation parameters, such as weather and time, the atmospheric transmittance is calculated. The scenario program can select the background and target trajectory, and then, SE-RAY-IRsynthesizes the IR sequences using the ray tracing method. Figure 22 shows the APS scenario and corresponding synthesis IR image (Set4). Two targets (one is the real target, the other is a decoy) were inserted, and the target distance was 1.23 km.

**Figure 23:**
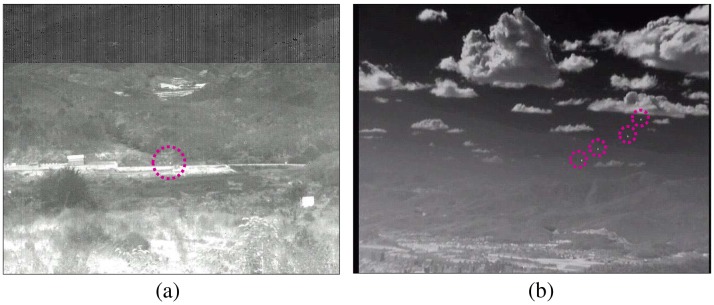
Real target sequence: (**a**) incoming Metis-Mmissile; (**b**) maneuvering multi targets (F-15K).

The first real sequence contains a real antitank missile (Metis-M) incoming near the IR camera (Set5: Cedip, long-wave IR, 120 Hz, target distance 1.5 km), as shown in Figure 23a. The next real sequence consists of four F-15Kswith dynamic motion in strong cloud clutter (Set6).

The detection rate (DR) and false alarm rate (FAR) defined in Equations (14) and (15) were used as the comparison metrics. If the distance between a ground truth and a detected position is within a threshold (e.g., three pixels), then the detection is declared to be correct. The DR represents how many correct targets are detected among the true targets, and the FAR represents how many false targets are detected per frame on average.

(14) $$DR[\%]=\frac{\text{Number of correctly}‐\text{detected targets}}{\text{Number of true targets}}$$

(15) $$\text{FAR}[\#]=\frac{\text{Number of incorrectly}‐\text{detected targets}}{\text{Number of frames}}$$

**Figure 24:**
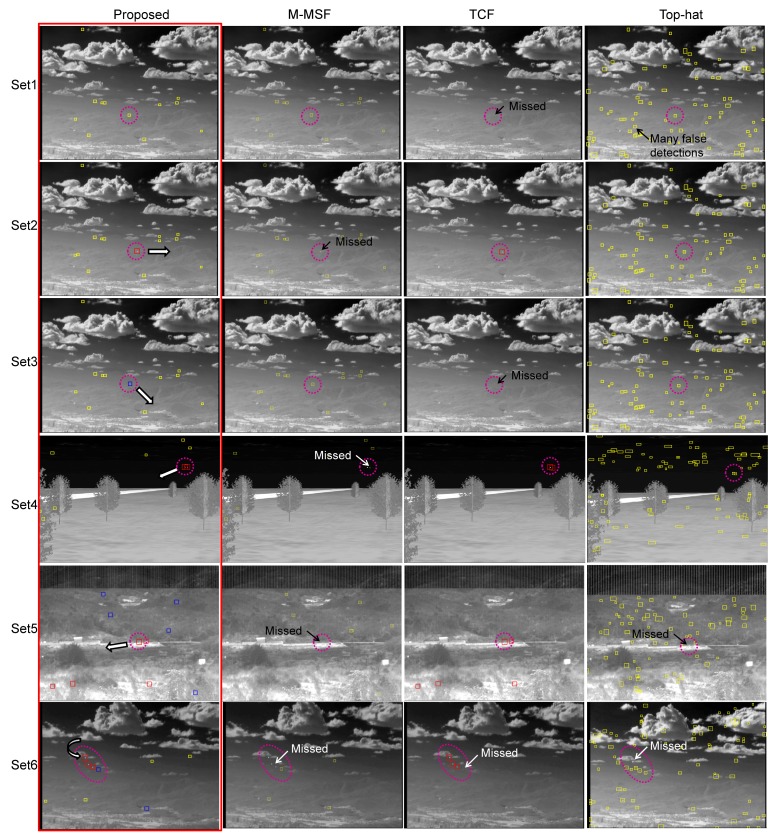
Performance comparison examples of the small target detection methods for the six kinds of test sets. The dotted circles represent the ground truth, and the rectangles represent detection results.

**Figure 25:**
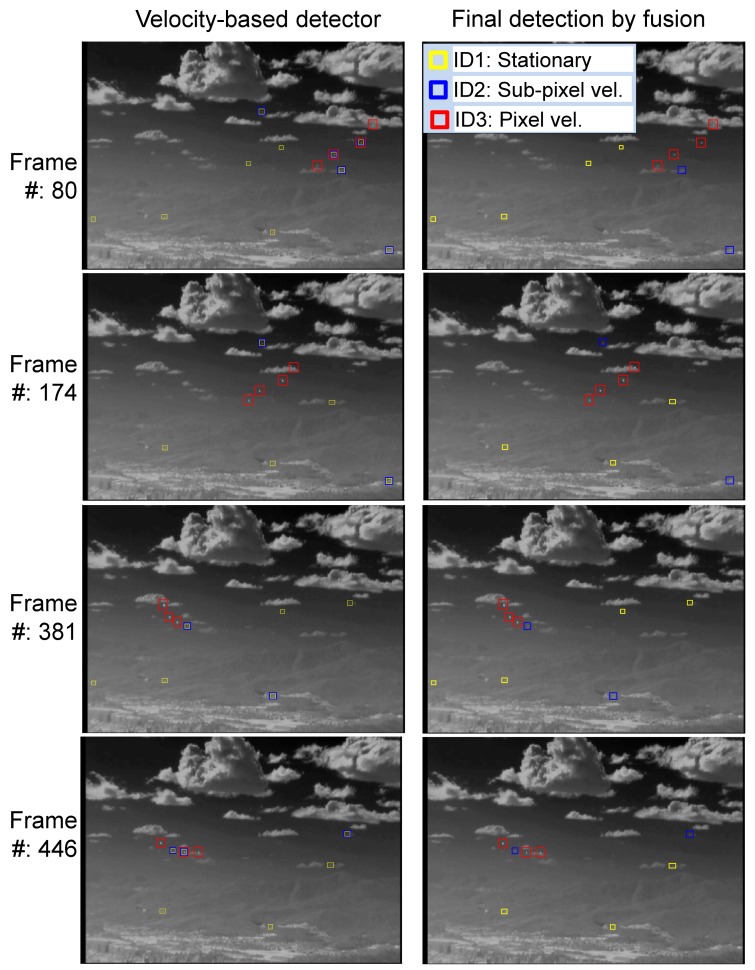
Effects of the proposed target detection system on the test Set6.

The proposed method was evaluated in terms of the target detection performance for the above mentioned scenarios. M-MSF [10], TCF [37] and top-hat [9,60,61] were used as the baseline methods, and the proposed fusion method was compared. The top-hat method was well studied and showed good performance in terms of the small target detection problem. The recent method, BMVT [21], was tested on our highly cluttered dataset and showed a poor result. Therefore, the method was excluded in the evaluation. The related parameters were set as follows: The buffer size of *TCF_long_* was set to 25, and that of *TCF_short_* was set to five. *Th_low_* in M-MSF was set as 10. The *TH_CFAR_* in M-MSF was set to six. The *TH_sub_*_−_*_pixel_* in the *TCF_long_* was set to 10. These parameters were tuned to achieve the best results, and the same values were used for each detection method. In the top-hat, the same parameters (*Th_low_* = 10, *TH_CFAR_* = 6) were used, except the structured element size (3 × 3). Table 1 summarizes the statistical performance comparisons of the proposed method, M-MSF (spatial filter) [10], TCF (temporal filter) [37] and top-hat [61] in terms of the detection rate and the false alarm rate. According to the results, the proposed detection method produces the best detection rate with moderate false alarms. The baseline method, top-hat, showed a relatively moderate detection rate. However, it generated many false detections per frame, such as 71–118 (#/fr), depending on the background clutter. Figure 24 provides examples of the sample test results for each dataset. Set1 contains only an LOS incoming target whose image velocity is zero (pixels/frame). As can be seen, the proposed method detected the target correctly, and the TCF missed it. The top-hat method generated many false detections around cloud and ground. Set2 contains a passing-by target, whose motion is right in the image. The M-MSF fails to detect the target due to background (mountain) clutter. Set3 has an incoming missile whose initial velocity is zero and increases slowly. Therefore, the TCF missed the initial target. Set4 has two incoming missiles: one is a decoy, and the other is the true target. M-MSF failed to detect, because a close target increased the threshold. Set5 is an incoming real Metis-M missile sequence. The M-MSF and top-hat failed to detect it, because the target is located in strong ground clutter. Set6 has four maneuvering targets. The proposed detector showed complementary detection performance in this case. The top-hat missed a strong target on the edge of cloud clutter. Figure 25 shows the effects of the proposed velocity-based detector and the final detection by threat-priority-based fusion. Note that the proposed detection scheme can not only provide a high detection rate, but also identify the target attributes, such as the stationary target, sub-pixel velocity target and pixel velocity target. This information can be useful to a tracking system.

**Figure 26:**
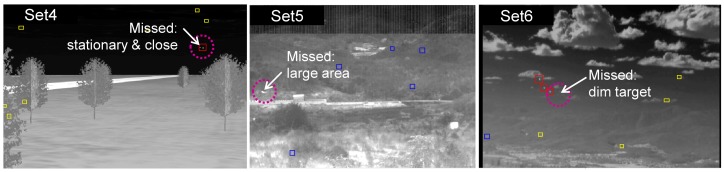
Examples of failure cases of the proposed method.

Although the proposed target detection method showed powerful detection capability, it misses some targets, as shown in Figure 26. In the first example, the missed target is stationary and close to a strong target. The stationary target detector uses H-CFAR, and the neighboring target belongs to background clutter, which makes a large standard deviation. Therefore, the stationary target was missed due to the reduced signal-to-clutter ratio (SCR). In the second case, the fast moving Metis-M missile was missed due to the large target size (80 pixels). We limited the maximum size to 60 pixels. In the last example, the proposed method could not detect the dim target. It can be possible to detect the dim target if the detection threshold is decreased. However, this approach will produce a number of false detections.

## 6. Conclusions

The next generation active protection system (NG-APS) requires infrared-based small target detection methods with a high detection capability at the lowest computational cost. Conventional state-of-the-art small target detectors based on either a spatial filter or temporal filter have their own advantages and disadvantages. According to the analysis of incoming targets in terms of the image size and motion, they showed a point-like size and varying image velocity, such as stationary at the firing time, slow motion at mid-course and fast motion at the final stage. One filter cannot detect those targets. The key idea is to use three kinds of velocity filters: stationary, sub-pixel velocity and pixel velocity. The stationary target detector uses only strong spatial shape information. The sub-pixel velocity target detector uses both the spatial cue and temporal motion cue, because the sub-pixel velocity targets have very small signal changes that cause false alarms in temporal noise. This problem is overcome using spatial information. The last pixel-velocity target detection uses only a temporal cue, because fast moving targets are normally located near the camera, which leads to a strong signal change in the temporal domain. The three kinds of detection results are fused using the threat-priority-based method. As validated by six sets of experiments, it can achieve the highest detection rate in various target motion scenarios. Furthermore, the proposed detection system can provide target attribute information, such as the motion property. The simplicity of the algorithm with a powerful detection capability highlights its real-time military applications for NG-APS and other surveillance problems.

## Figures and Tables

**Table 1:** Statistical performance comparisons of the small infrared target detection methods (DR: detection rate; FAR: number of false alarms per frame; PE: position error).

**Method**	**Perf. Measure**	**Set1: Syn LOS**	**Set2: Syn Passing-By**	**Set3: Syn Incoming**	**Set4: Syn OKTAL-SE**	**Set5: Real Metis-M**	**Set6: Real F-15 Multi**
Proposed	DR (%)	**100.0** (120/120)	**100** (33/33)	**100** (110/110)	**97.4** (224/230)	**99.1** (114/115)	**99.3** (3178/3200)
	FAR (#/fr)	11 (1320/120)	13 (429/33)	12 (1320/110)	7 (805/115)	7.1 (821/115)	5 (4055/811)
M-MSF [10]	DR (%)	86.7 (104/120)	3 (1/33)	89.1 (98/110)	0 (0/230)	0 (0/115)	92.2 (2915/3200)
	FAR (#/fr)	11 (1320/120)	13 (429/11)	12 (1320/110)	7 (805/115)	6.7 (776/115)	5 (4055/811)
TCF [37]	DR (%)	10.0 (12/120)	100 (33/33)	96.3 (106/110)	97.4 (224/230)	99.1 (114/115)	98.7 (3161/3200)
	FAR (#/fr)	0 (0/120)	0 (0/33)	0 (0/110)	0 (0/115)	0.4 (45/115)	0.003 (2/811)
Top-hat [61]	DR (%)	97.5 (117/120)	100 (33/33)	94.5 (104/110)	96.9 (223/230)	35.6 (41/115)	80.6 (2580/3200)
	FAR (#/fr)	116 (13,920/120)	118 (3894/33)	117 (12,870/110)	92(10,582/115)	71 (8165/115)	76 (61,316/811)
